# Efficiency and complications in root canal retreatment using nickel titanium rotary file with continuous rotation, reciprocating, or adaptive motion in curved root canals: a laboratory investigation

**DOI:** 10.1186/s12903-023-03610-x

**Published:** 2023-11-16

**Authors:** Benjaporn Tantiwanichpun, Sirinya Kulvitit

**Affiliations:** 1 Department of Operative Dentistry, Faculty of Dentistry, Chulalongkorn University, Bangkok, 10330 Thailand; 2 Center of Excellence in Genomics and Precision Dentistry, Faculty of Dentistry, Chulalongkorn university, Bangkok, 10330 Thailand

**Keywords:** Gutta percha, Root canal filling removal, Endodontic retreatment, Broken instrument, Perforation, NiTi rotary file, Micro-computed tomography, Continuous rotation, Reciprocation, Adaptive motion

## Abstract

**Background:**

It is currently unknown whether rotary file motion affects the best outcome of root canal retreatment. This experimental study compared the efficacy, efficiency, and complications of single-use NiTi rotary files using continuous rotation, reciprocating, and adaptive motions in root canal filling removal in curved root canals. Reciproc blue R25 was used with reciprocating motion (RB), VDW.ROTATE retreatment files with continuous rotation (VR), and ProTaper NEXT X2 with continuous rotation (PTNc) or adaptive motion (PTNa).

**Methods:**

Forty mesial root canals of extracted mandibular first and second molars with an angle of curvature between 20°–40° and a radius of curvature between 5 and 10 mm were collected. The specimens were instrumented and obturated with gutta-percha and AH Plus sealer using the continuous wave of condensation technique. The specimens were randomly divided into 4 retreatment groups (n = 10), RB, VR, PTNc, and PTNa. The percentage of root canal filling removal in each group was analyzed using Micro-Computed Tomography (µCT). The motor running time, total time, root canal complication, and instrument complication were recorded and statistically analyzed (*p*-value < 0.05).

**Results:**

The pre-operative root canal curvature and root canal filling volume were comparable among groups. The percentage of root canal filling removal from the whole canal in the PTNc, RB, PTNa, and VR group was 98%, 96%, 95%, and 93%, respectively. A significant difference was observed between the PTNc and VR groups for the whole canal and the apical-third part. The motor running time and total time were significantly different between the groups. Instrument fracture was observed at 40% in the VR and 20% in the PTNa group, but none in the RB and PTNc groups.

**Conclusions:**

The ProTaper NEXT X2 with continuous rotation and RB files can be used with high efficacy and efficiency in curved root canal retreatment. Continuous rotation is more efficacious and efficient than adaptive motion when using the NiTi rotary file. Single file retreatment can be used in small canals with high efficacy, cost-effectiveness, and less time consumption.

## Introduction

Non-surgical root canal retreatment (NS-ReTx) is the treatment of choice for both endodontic failure and endodontic success cases. NS-ReTx is performed in endodontic failure cases to remove as much intraradicular infection as possible to allow periapical lesion healing [1]. Furthermore, NS-ReTx is used in endodontic success cases that have inadequate root canal obturation and a new restoration is needed, to prevent periapical lesion development after fixing the new restoration [2, 1].

The most important step in root canal retreatment is removing the root canal filling. Removing the existing root canal filling is the critical step to regain access to the apical foramen and allow for sufficient cleaning and disinfection of the root canal system [1, 3]. There are several considerations for root canal filling removal: root canal anatomy, possible complications, and type of root canal filling [2].

Gutta-percha, a commonly used root canal filling material, is a semisolid substance that can be removed during retreatment procedures. Factors affecting gutta-percha removal include its condensation density, obturation length, and root canal shape [1]. Previously, rotary instruments were primarily recommended for gutta-percha removal in straight root canals, while curved root canals required gutta-percha dissolution with solvents and removal using hand files. However, the advent of Nickel-titanium (NiTi) rotary instruments in endodontics has expanded the applicability of rotary instruments to curved root canals.

The efficacy of root canal filling removal is represented by the cleanliness of the root canal after the removal procedure and the ability to remove the root canal filling without causing any root canal complications, such as ledges and perforations. Efficiency is the ability to remove the root canal filling using less time and fewer rotary files.

The reported efficacy and efficiency of each NiTi rotary system is different due to the different methodologies between studies. Many studies evaluated root canal wall complications, NiTi rotary file complications, cleanliness, and time consumption. Furthermore, most previous studies used NiTi rotary files more than once for retreatment procedures that might not show the true efficacy and efficiency of the file [4–6]. This is because the stress that builds up in the material during retreatment may decrease the rotary files’ efficacy and efficiency [7].

There were differences in the efficacy and efficiency of root canal filling removal in curved canals using rotary files with different motions. Continuous rotation is a continuous clockwise rotation of the rotary file, while reciprocating motion is a clockwise and counterclockwise rotation of the rotary file that completes one cycle of 360º rotation every three cycles. Adaptive motion is a continuous clockwise rotation in a stress-free situation, and switches to a reciprocating motion when under stress [8].

Studies comparing the different file motions have generated disparate results. Some studies found similar efficacy and efficiency between using continuous rotation and reciprocating motions in root canal filing removal in curved canals [9–11]. However, other studies found that reciprocating motion significantly removed 2-fold more root canal filling than continuous rotation [12]. Furthermore, another study found that continuous rotation removed more root canal filling and faster than reciprocating motion [13]. Moreover, studies demonstrated that adaptive motion significantly removed 6–10% more root canal filling than continuous rotation [8, 14]. However, rotary files using adaptive motion failed to retain their original shape, including 50% deformation and 50% fracture [15].

Despite the efficacy and efficiency of NiTi rotary instruments in curved root canal retreatment, procedural accidents, such as broken NiTi rotary instruments during curved root canal retreatment have been reported [4, 9, 16]. When instruments separate, more than half of those occur during retreatment procedures in a severely curved root canal [17].

It is currently unknown whether Twisted file adaptive (TF adaptive) files or adaptive motion leads to instrument complications during retreatment. Furthermore, there has been no report comparing the three motions in curved root canal retreatment using rotary files. Therefore, the efficacy of which NiTi rotary file motion can retreat a curved root canal without root canal or rotary file complications is currently unknown.

The aim of this experimental study was to compare the efficacy, efficiency, and complications of single-use NiTi rotary files using continuous rotation, reciprocating, and adaptive motions in root canal filling removal in curved root canals. The rotary files used in this study were produced by heat-treated NiTi with a non-cutting tip design. Reciproc Blue files were blue wire heat-treated NiTi with an s-shaped cross-section, VDW.ROTATE retreatment files were specifically heat-treated NiTi with an s-shaped cross-section and ProTaper NEXT files were M-wire heat-treated NiTi with an off-center rectangular cross-section.

The null hypotheses of this study are that there is no significant differences in the percentage of root canal filling removal, time consumed and rotary file or root canal wall complications during curved root canal retreatment between these instruments using different motions.

## Materials and methods

This experimental study was approved by the Ethics Committee.

### Sample collection

Freshly extracted human teeth were collected. The sample size calculation was based on Crozeta BM. 2016 [8], using the G*Power 3.1 program with a type I error of 0.05 and 0.8 power. The calculation indicated that an adequate sample size was 40 canals (10 per test group). Due to the nature of the study, there were no negative control specimens. However, the specimens retreated by Reciproc® *blue* R25 files served as a positive control. This is because the Reciproc® *blue* file manufacturer states that they can be used as retreatment files and several studies found high efficiency and safety when using these files for root canal retreatment [5, 18].

Twenty extracted mandibular first and second molars with two mesial canals were collected and disinfected in 10% formalin. The inclusion criteria were teeth without prior endodontic treatment, without calcification, no resorption, closed apex, no root caries, and no cracks. Buccolingual and mesiodistal radiographs were used to determine the root canal curvature. The specimens with separate mesial root canals (type IV Vertucci’s classification) [19] with curvatures between 20°–40° and a radius of curvature between 5 and 10 mm were included.

### Specimen preparation

The specimens were prepared by the single operator (B.T.) from decoronation to root canal obturation. The tooth crown was removed using a round-ended taper diamond bur to obtain a standard root length of 13 mm. The specimens underwent coronal flaring using SX rotary files (Dentsply Maillefer, Ballaigues, Switzerland). The glide path was created using no.10 and 15 K-files. The working length was set at 1 mm short from the apical foramen. Teeth with an initial apical file greater than 25 were excluded from the experiment. The specimens were then shaped with ProTaper NEXT X1 (tip size 17; variable taper; Dentsply Maillefer) followed by ProTaper NEXT X2 (tip size 25; variable taper; Dentsply Maillefer) rotary files at 300 rpm and 2.5 Ncm torque until reaching the working length. The root canal was irrigated with 2.5% sodium hypochlorite (NaOCl) during instrumentation, and received a final rinse with 10 ml 17% ethylenediamine tetraacetic acid (EDTA) followed by 10 ml 2.5% NaOCl. The canals were dried with paper points.

The dried canals were obturated with ProTaper NEXT X2 matched cones (Dentsply Maillefer) and AH Plus, epoxy resin-based sealer (Dentsply Maillefer) using the continuous wave of condensation technique. The gutta-percha cone was coated with sealer and seated with tug back. After down-packing the gutta percha using Fast-Pack PRO (Eighteeth, Changzhou, China) to 5 mm from the working length, the canal was backfilled with warm regular gutta-percha (Eighteeth) to the canal orifice using Fast Fill (Eighteeth) and BL-S Kondenser (B&L Biotech, Fairfax, VA, USA). After obturation, the specimens were radiographed to determine the root canal filling quality and re-calculate the root canal curvatures.

### Pre-micro-computed tomography (µCT) analysis of the root canal filling volume

To measure the root canal filling volume, the specimens were scanned using a Micro-CT scanner 35 SCANCO MEDICAL (CH-8306 Bruettisellen, Switzerland) using µCT Tomography V.6.4 software, voxel size 18.5 μm, 231 slices, 70 kVp, 114 µA, and 8 W. µCT Tomography V 6.6 software was used to determine the root canal filling volume. The Pre-Micro-CT data were collected, and the whole canal filling volume and the coronal-, middle- and apical-third filling volumes were calculated. The root canal filling volume was reported in mm^3^.

### Root canal retreatment

The prepared specimens were randomly divided into four experimental groups according to the rotary file system and motion used (n = 10). One independent researcher, who did not know the aim of the study, randomly selected the specimens that had been placed in an envelope and assigned them to one of the groups. The root canal retreatment was done using a dental operating microscope (DOM) (Zeiss, Munich, Germany) by the single operator (S.K.), an 8-years’ experienced endodontist, to minimize the differences in the procedural technique between the experimental groups. The coronal 3 mm of the root canal filling was removed by a no.3 Gates-Glidden drill (Kerr Dental, Orange, CA). After the Gates-Glidden drill created the path, the rotary file was used for penetrating and removing the root canal filling.

#### Group 1

Reciproc® *Blue* system (RB).

The retreatment procedure was performed with the Reciproc Blue R25 files (tip size 25; variable taper; VDW, Munich, Germany) using the Endodontics motor X-SMART IQ™ (Dentsply Maillefer) set in reciprocating mode. The motor was controlled by The DENTSPLY ENDO iQ Application. During the procedure, the application recorded the total time, motor running time, and maximum torque of each specimen. The instrument was advanced apically using an in-and-out pecking motion with an amplitude of approximately 3 mm. Gentle apical pressure was applied with a brushing action against the lateral walls according to the manufacturer’s instructions. After every three pecking motions, the instrument was removed from the canal and cleaned with sterile gauze.

#### Group 2

VDW.ROTATE retreatment system (VR).

The retreatment procedure was performed with the VDW.ROTATE retreatment files (tip size 25; 5% taper; VDW) using the Endodontics motor X-SMART IQ™ set in continuous rotation mode at 500 rpm and 3.5 Ncm torque. The retreatment technique was the same as that described for Group 1.

#### Group 3

ProTaper NEXT X2 continuous rotation (PTNc).

The retreatment procedure was performed with the ProTaper NEXT X2 files (tip size 25; variable taper) using the Endodontics motor X-SMART IQ™ (Dentsply Maillefer) set in continuous mode at 500 rpm and 3.5 Ncm torque. The retreatment technique was the same as that described for Group 1.

#### Group 4

ProTaper NEXT X2 adaptive motion (PTNa).

The retreatment procedure was performed with the ProTaper NEXT X2 files using the Endodontics Elements Motor (SybronEndo, Glendora, CA) set in adaptive mode. The retreatment technique was the same as that described for Group 1. Because TF adaptive ML1 files (tip size 25; taper 0.08) deformed in our pilot study and a previous study (Ozyurek & Demiryurek 2020), they were not utilized in this study.

In all groups, after the NiTi rotary file reached the working length and the time was recorded, the operator (S.K.) continued using the file to completely remove the root canal filling until the root canal was clean as seen using the DOM and no filling material was observed on the instrument flutes. If the file was deformed at any stage of the root canal retreatment, the retreatment procedure continued until the working length was reached and root canal was clean as seen using the DOM and no filling material was observed on the instrument flutes or until the file was broken. The dental operating microscope magnification was set at 1.6 (13.6-fold magnification) while performing root canal retreatment with a rotary file. It was then adjusted to 2.5 (21.25-fold magnification) for inspecting the remaining root canal filling on the root canal wall. The specimens with broken files were not included in the Post-Micro-CT analysis, because the remaining root canal filling volume was assumed to be overestimated in these specimens.

Each NiTi rotary file was used only once for each canal. When the retreatment procedure was completed, the canals were irrigated with 10 ml 2.5% NaOCl and then dried with paper points. The total time and motor running time were recorded by The DENTSPLY ENDO iQ Application in groups 1–3, or by a digital clock in group 4. The total time included the motor running time, file cleaning time, and irrigation time.

### Post-micro-computed tomography (µCT) analysis of the root canal filling volume and data collection

To measure the root canal filling volume after the retreatment procedures, the specimens were scanned by a Micro-CT scanner 35 SCANCO MEDICAL (CH-8306 Bruettisellen, Switzerland) using the same parameters as in the Pre-Micro-CT analysis. The Post-Micro-CT analysis data were collected and the root canal filling volume in the whole canal, coronal-, middle-, and apical-thirds were calculated. The root canal filling volume was reported in mm^3^.

During the Post-Micro-CT analysis, the operator (B.T.) knew the specimen number, but not which type of NiTi rotary file was used. The Micro-CT volumes measurements before and after the retreatment procedures were conducted by the same operator and the volumes were evaluated twice to determine the intraobserver reliability, using the intraclass correlation coefficient (ICC) [20]. The observer re-evaluated 20% randomly selected root canal filling volumes at least 1 week later.

The root canal complications (ledge or perforation) and instrument complications (deformation or separation) during the retreatment procedures were collected. All rotary files used in the experiment were inspected under DOM and captured. The percentage of root canal filling removal between each area of each specimen was calculated. In addition, the retreatment time, i.e., total time and motor running time, were analyzed. Superimposition and reconstruction of the pre-µCT and post-µCT images of representative samples were performed using the Materialise Mimics Version 25.0.1.583 software (Leuven, Belgium).

### Statistical analyses

The IBM® SPSS® Statistics Version 28 (IBM Corp.©) was used for the statistical analyses. The Shapiro-Wilk test was used to evaluate the normality of the data. If the data had a normal distribution (Shapiro-Wilk, P > 0.05), the difference between groups was compared using One-way ANOVA followed by Bonferroni test. Whereas, if the data had a skewed distribution (Shapiro-Wilk, P < 0.05), the difference between groups was compared using the Kruskal-Wallis test followed by Dunn’s test. P values less than 0.05 were considered significant for all tests.

## Results

The pre-operative canal characteristics between the groups were analyzed. The mean values of the angle and radius of curvature in the mesio-distal and bucco-lingual aspects were similar between the groups (Table 1). The angle of the root canal curvature in the mesio-distal direction before retreatment ranged from 21.95°–22.25°. The radius of the curvature in the mesio-distal direction before retreatment ranged from 7.60 to 7.96 mm. The angle of the root canal curvature in the bucco-lingual direction before retreatment was between 8.1°–9.8°. The radius of the curvature in the bucco-lingual direction before retreatment was between 10.88 and 12.24 mm. Moreover, the mean pre-operative root canal filling volume was similar between groups (Table 2). The intraobserver reliability based on an ICC of 0.99 indicated excellent reliability [20].

**Table 1:** The mean values of the angle and radius of the root canal curvature in each group

	Mean angle of curvature of samples		Mean radius of curvature of samples	
	**Mesio -Distal** ^A^	**Bucco - Lingual** ^A^	**Mesio -Distal** ^A^	**Bucco - Lingual** ^B^
Group 1 (RB)	21.95 ± 2.36	9.40 ± 6.70	7.71 ± 1.46	10.88 ± 5.41
Group 2 (VR)	22.15 ± 2.26	9.80 ± 7.71	7.96 ± 0.90	11.23 ± 6.19
Group 3 (PTNc)	22.25 ± 1.84	8.30 ± 7.38	7.83 ± 1.61	11.74 ± 6.01
Group 4 (PTNa)	22.05 ± 1.98	8.10 ± 6.92	7.60 ± 1.23	12.24 ± 5.64

^A^From the ANOVA test (p < 0.05)

^B^From the Kruskal-Wallis test (p < 0.05)

**Table 2:** The mean values of the Pre-Micro-CT volume in each group in the whole canal, coronal-, middle-, and apical-thirds of the root canal

	Volume of filling material			
	**Whole canal** ^A^	**Coronal 1/3** ^B^	**Middle 1/3** ^A^	**Apical 1/3** ^A^
Group 1 (RB)	5.07 ± 0.95	3.01 ± 0.52	1.48 ± 0.39	0.58 ± 0.15
Group 2 (VR)	4.93 ± 1.18	3.00 ± 0.77	1.36 ± 0.33	0.56 ± 0.13
Group 3 (PTNc)	4.68 ± 0.80	2.81 ± 0.63	1.35 ± 0.22	0.52 ± 0.10
Group 4 (PTNa)	5.08 ± 1.08	3.07 ± 0.74	1.45 ± 0.35	0.56 ± 0.17

^A^From the ANOVA test (p < 0.05)

^B^From the Kruskal-Wallis test (p < 0.05)

**Fig. 1:**
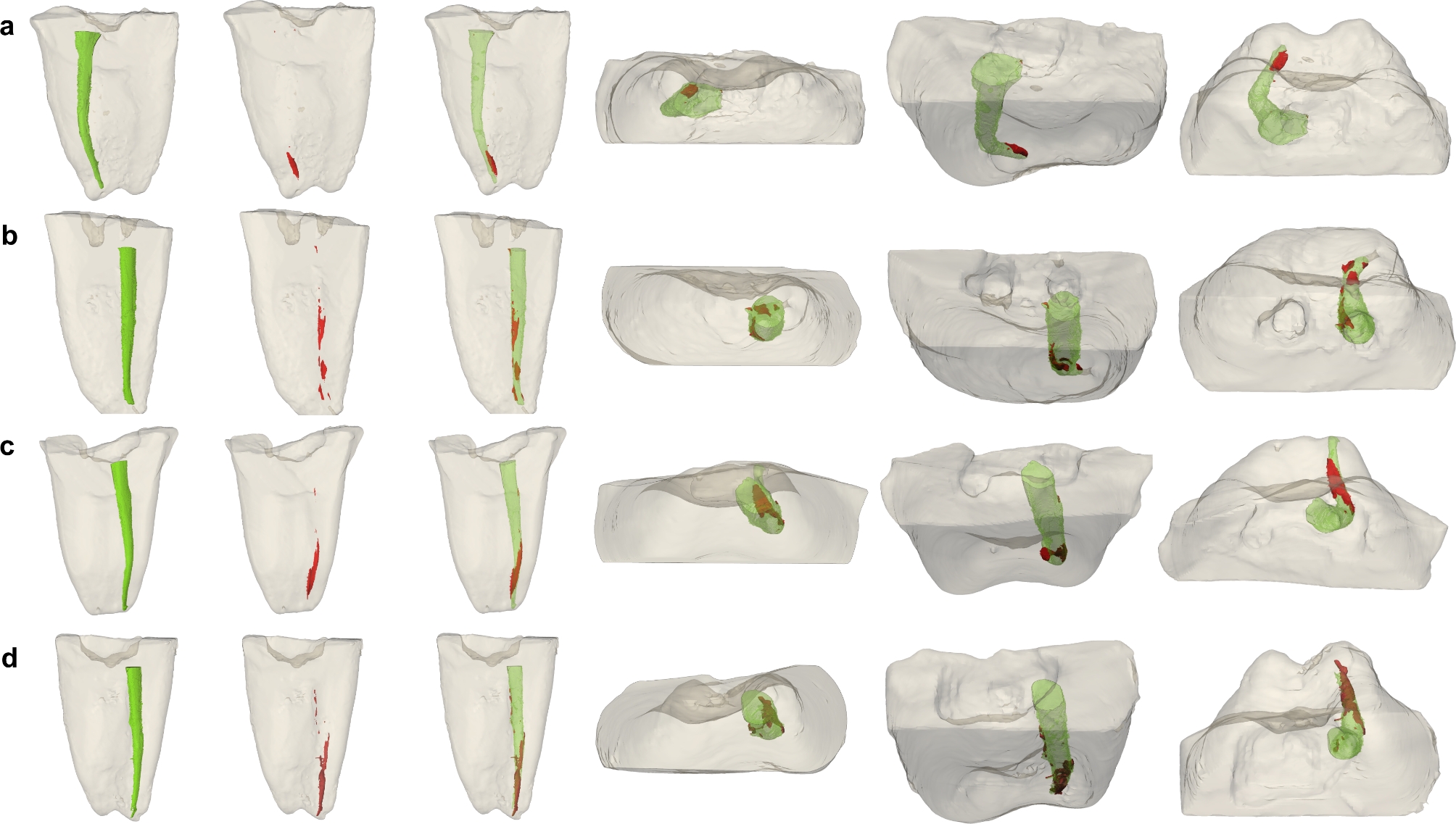
Superimposition and reconstruction of the pre-µCT and post-µCT images of representative samples in each group. From left to right: Obturation material (green), remaining obtruration material post-retreatment (red), superimposed image, occlusal view, occluso-mesial view, and occluso-distal view. **(a)** PTNc, **(b)** RB, **(c) **PTNa, and **(d)** VR.

The percentages of root canal filling removal were significantly different between some groups. In the whole canal analysis, the percentage of root canal filling removal in the PTNc, RB, PTNa, and VR group was 98%, 96%, 95%, and 93%, respectively. A significant difference was found between the PTNc and VR groups (mean difference (MD) = 5.4; *p* = 0.006; 95% confidence interval (CI): 1.22 to 9.52). The coronal- and middle-third analyses demonstrated that the percentage of root canal filling removal was similar between groups. The apical-third analysis indicated that the percentage of root canal filling removal in the PTNc group, RB group, PTNa group, and VR group was 93.22%, 90.43%, 80.04%, and 56.32%, respectively. A significant difference was again found between the PTNc and VR groups (MD = 36.9; *p* = 0.004; 95% CI: 9.28 to 64.52). Moreover, there were significant differences in the percentage of root canal filling removal in the coronal-, middle-, apical-thirds, and whole canal analysis between the groups (Table 3). The superimposition and reconstruction of the pre-µCT and post-µCT images of representative samples in each group are seen in Fig. 1.

**Table 3:** The mean percentage of the root canal filling removal in each group in the whole canal, coronal-, middle-, and apical-thirds of the root canal

	Mean percentage of volume reduction			
	**Whole canal** ^A^	**Coronal 1/3** ^A^	**Middle 1/3** ^A^	**Apical 1/3** ^A^
Group 1 (RB)	96.93 ± 1.52^a^	97.98 ± 1.66^b^	97.66 ± 2.60^c^	90.43 ± 9.18^abc^
Group 2 (VR)	93.14 ± 4.86^ay^	98.45 ± 1.21^b^	97.43 ± 2.64^c^	56.32 ± 35.31^abcz^
Group 3 (PTNc)	98.51 ± 0.95^ay^	99.03 ± 0.70^b^	99.69 ± 0.43^c^	93.22 ± 7.39^abcz^
Group 4 (PTNa)	95.89 ± 3.70^a^	98.14 ± 2.34^b^	97.45 ± 3.22^c^	80.04 ± 21.63^abc^

^A^From the ANOVA test followed by Bonferroni test (p < 0.05)

Same superscript lowercase letters indicate a significant difference between groups (^abc^ for rows and ^yz^ for columns)

Sample with broken rotary files not included

The motor running time until reaching the working length and until complete removal using a DOM in the VR group was significantly shorter compared with the RB (MD = 47.7; *p* < 0.001; 95% CI: 26.64 to 68.69 for time until reaching the working length and MD = 61.2; *p* < 0.001; 95% CI: 31.47 to 90.93 for time until complete removal) and PTNa groups (MD = 49.3; *p* < 0.001; 95% CI: 27.30 to 71.28 for time until reaching the working length and MD = 67.0; *p* < 0.001; 95% CI: 35.91 to 98.09 for time until complete removal) (Table 4). Furthermore, the total time until reaching the working length and until complete removal using a DOM in the VR group was significantly shorter than the RB (MD = 92.7; *p* < 0.001; 95% CI: 37.02 to 148.38 for time until reaching the working length and MD = 166.8; *p* < 0.001; 95% CI: 41.64 to 292.03 for time until complete removal) and PTNa (MD = 130.4; *p* < 0.001; 95% CI: 72.14 to 188.61 for time until reaching the working length and MD = 208.6; *p* < 0.001; 95% CI: 77.65 to 339.52 for time until complete removal) groups. In addition, the total time until reaching the working length, and until complete removal in the PTNc group was significantly shorter compared with the PTNa group (MD = 81.9; *p* < 0.001; 95% CI: 30.83 to 133.12 for time until reaching the working length and MD = 158.6; *p* < 0.001; 95% CI: 43.55 to 273.55 for time until complete removal) (Table 4).

**Table 4:** The mean total time and motor running time in the retreatment procedure in each group

	Mean total time		Mean motor running time	
	**Reach WL** ^A^ **(sec)**	**Complete removal** **under DOM** ^A^ **(sec)**	**Reach WL** ^A^ **(sec)**	**Complete removal** **under DOM** ^A^ **(sec)**
Group 1 (RB)	117.20 ± 26.25^a^	256.00 ± 84.01^a^	62.00 ± 14.41^a^	91.20 ± 20.68^a^
Group 2 (VR)	24.50 ± 10.29^a,b^	89.17 ± 54.97^a,b^	14.33 ± 6.44^a,b^	30.00 ± 11.90^a,b^
Group 3 (PTNc)	72.90 ± 28.82^c^	139.20 ± 56.84^c^	35.90 ± 12.85	62.00 ± 14.29
Group 4 (PTNa)	154.88 ± 64.93^b,c^	297.75 ± 127.22^b,c^	63.63 ± 19.53^b^	97.00 ± 29.43^b^

^A^From the ANOVA test followed by Bonferroni test (p < 0.05)

Same superscript lowercase letters indicate a significant difference between groups

Samples with broken rotary files not included

**Fig. 2:**
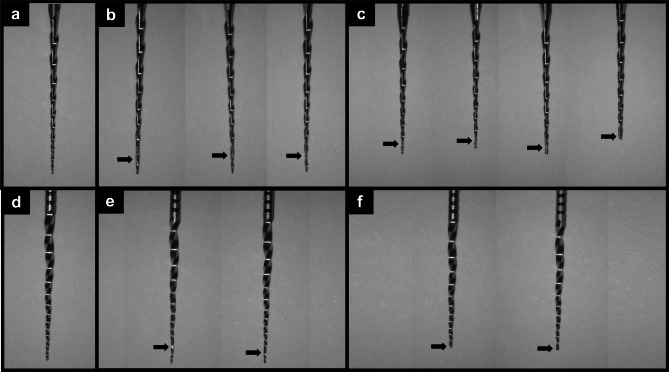
Rotary files used in retreatment procedure captured under a DOM set at 1.6 magnification (13.6-fold magnification). **(a)** pre-experiment: new VR file, **(b)** post-experiment: three VR files with only deformations, **(c)** post-experiment: four VR files with deformations and separations, **(d)** pre-experiment: new X2 file, **(e)** post-experiment: two files in the PTNa group with only deformations, and **(f)** post-experiment: two files in the PTNa group with deformations and separations. All images were captured by same DOM with controlled magnification and setting. Arrows indicate file deformation

During the retreatment procedure, the maximum torque was not reached, and root canal complications were not observed in any specimen. However, instrument complications were observed in the VR and PTNa groups, but none in RB and PTNc groups. In the VR group, 30% of the instruments were deformed and 40% of the instruments were deformed and separated. Moreover, 20% of the instruments had deformations and 20% of the instruments had deformations and separations in the PTNa group (Fig. 2).

## Discussion

The present study compared the efficacy and efficiency of different NiTi rotary instruments using different motions in the retreatment procedures in curved root canals. We found that percentage of root canal filling removal, time consumed, and complications were significantly different between the file groups. Based on these results, the null hypotheses were rejected.

The angle and radius of curvature of the specimens, and pre-operative root canal filling volume were similar between groups. The specimens were prepared by a single operator using the same technique. These aspects suggested that the results of the present study were not affected by the differences in individual root canals or operators.

In the present study, the angle of curvature in the original root canals ranged from 21°–29° with a mean of 23.5°. This corresponds to the average angle of curvature in mesial root canals of mandibular molars (23°–27°) [21, 22]. Thus, our specimens can be considered as the typical mesial root canals in mandibular molars that would be encountered in the clinic.

Retreatment in curved root canals studies usually reported the original canal curvature of their specimens. However, a previous study found that the angle of curvature in the root canal was significantly reduced up to 3% after instrumentation [23]. The angle of curvature in the root canals in the present study was also reduced 5.9% after instrumentation. Therefore, the present study reported the angle of curvature in the root canals after instrumentation and obturation that were between 21.95°–22.25° with a mean of 22.1°.

The present study found that none of the NiTi rotary systems completely removed the root canal filling. The percentage of root canal filling removal in the whole canal ranged from 93 to 98%. A significant difference in the root canal filling removal percentage was found between the PTNc and VR groups. However, the percentage of root canal filling removal in the whole canal in the PTNc group was only 5% significantly higher than the VR group. Although the overall percentage difference is small, it mostly derives from the large differences found in the apical-third. The differences between the whole canal volumes are due to the differences in the apical-third volumes, which are large, but make up a small percentage of the overall root canal volume, thus do not have a significant impact on the total volume results. However, the differences in the apical-third might have a clinical impact.

The percentages of root canal filling removal in the coronal- and middle-thirds in this study were high at 97–99%. The ratio of the coronal- and middle-third volumes to the whole canal volume was 89%, therefore, the percentage of root canal filling removal in the whole canal ranged from 93 to 98%. The coronal- and middle-thirds of the curved root canals are usually straight and large, thus the rotary file can operate easily in these thirds. Our findings were higher than previous studies that found that the percentage of root canal filling removal in the coronal- and middle-thirds ranged from 78 to 96% [9, 18]. The differences might be caused by two factors. First, the previous studies’ specimens had a greater mean angle of curvature (35.5°–42.5°). Second, our study inspected the cleanliness of the root canal using a DOM during retreatment. Using DOM for identifying the remaining material on the root canal wall proved superior to radiographic examination. This superiority stems from the DOM’s ability to provide direct localization of the remaining material with the aid of magnification and illumination, especially in the straight part of the root canal, whereas radiographic examination is limited in precisely localizing the remaining material [24, 25]. Thus, we encourage the use of a DOM for inspecting the remaining root canal filling after retreatment.

The percentage of root canal filling removal in the apical-third ranged from 56 to 93% that was 6–42% significantly less than the coronal- and middle-thirds. These findings corresponded to those of previous studies in which the percentage of root canal filling removal in the apical-third was 68–84% [9, 18]. The significant difference in the percentage of root canal filling removal in the apical-third resulted from a 37% difference between the PTNc and VR groups. Because the apical part of the curved root canal is usually curved and small, the small apical part of the rotary file that can reach this area had difficulty removing the root canal filling. Therefore, the percentage of root canal filling removal in the apical part of the curved root canal can be an indicator for the efficacy of NiTi rotary files and motions.

The present study found that the motor running time in the VR group was approximately 1 min significantly faster than the RB and PTNa group. The motor running time represents the efficiency of the NiTi rotary files. Many previous studies used motor running time to compare the rotary systems [10, 26, 27]. This is because the motor running time reflects the time that the instrument is active inside the canal. However, the motor running time and total time in the experimental groups ranged from 30 to 97 s and 89–297 s respectively. The specimens required less than 5 min to perform the retreatment procedures, and the differences between the groups might not be clinically meaningful.

The retreatment procedures used in the present study caused no complications to the root canal walls, such as ledges and perforations. These data indicate that the NiTi rotary systems in this study followed the curvature of the root canal during retreatment. Furthermore, a study using Reciproc Blue R40 (#40.06) reported a maximum apical transportation only 0.1 mm from the original root canal at 1 mm from the working length, which may not affect canal cleaning and shaping [5].

The present study found instrument complications in the VR and PTNa groups. There were file deformations and separations in VR and PTNa groups. The complications in the PTNa group were most likely due to the adaptive motion, because there were no file complications in the PTNc group.

A broken instrument in a curved root canal can occur due to torsional failure and cyclic fatigue failure [28, 29]. Cyclic fatigue failure can result from rotary file movement in the curved root canal. For root canal retreatment, the penetration of NiTi rotary files in the root canal filling can lead to torsional preloading, and consequently lower cyclic fatigue resistance. Moreover, torsional failure can be caused by different motions of the rotary file. In mechanical instrumentation, clockwise rotation generates stress in the file as it penetrates the root canal dentin. In contrast, counterclockwise rotation helps release the stress generated in the rotary file. However, during retreatment, clockwise rotation generates stress in the file while it penetrates the root canal filling material and counterclockwise rotation generates additional stress on the other side of the rotary file as it penetrates the root canal filling on the other side. Therefore, reciprocating and adaptive motions have a greater likelihood of instrument fracture due to torsional failure based on their respective motion parameters than continuous rotation [30–32].

This study found no deformation or separation of the X2 with continuous rotation and RB files. These results correspond to a previous study that reported no instrument complications in the ProTaper NEXT group, however, there was one Reciproc (R25 #25/0.08) file that fractured [9]. In contrast, a previous study found two ProTaper NEXT X2 fractures during curved canal retreatment. These fractures might have occurred because the rotary file was used in three root canals before being replaced [4].

Although RB was exposed to torsional preloading from the reciprocating motion, we found that RB removed the root canal filling with high efficacy and efficiency, without any complications. RB is manufactured using a proprietary heat treatment that gives the instrument a blue color with improved flexibility and cyclic fatigue resistance. RB demonstrated a significantly higher cutting efficiency compared with Reciproc [33]. RB also had a higher cyclic fatigue resistance compared with Reciproc when evaluated using a dynamic cyclic fatigue testing device [34]. Because the taper, cross-sectional shape, and motion between RB and Reciproc were identical, the cutting efficiency and cyclic fatigue resistance of RB was the effect of the blue heat-treated alloy.

Our pilot study evaluated a retreatment protocol using TF adaptive files (ML1; tip size 25; 8% taper) in adaptive motion. In that study, the ML1 files were deformed and could not completely remove the root canal filling. Moreover, a previous curved root canal retreatment study reported that 100% of TF adaptive files using adaptive motion had defects after retreatment [15]. Therefore, the ML1 file was excluded from the present study and was replaced with X2 files in adaptive motion.

NiTi rotary files with a non-active tip are preferred in curved canal retreatment because they can follow the curvature and reduce root canal complications. However, the NiTi rotary files with a non-active tip had difficulty penetrating into the root canal filling and became deformed. Therefore, a Gates-Glidden drill was used to create the starting point into the root canal filling to allow the NiTi rotary file to penetrate the root canal filling [5, 13].

In the current study, after file deformation, the retreatment procedure continued until the rotary file reached the working length and the root canal was cleaned using a DOM and no filling material was observed on the instrument flutes or until the file was broken. In the VR and PTNa groups, the percentage of root canal filling removal and motor running time between the non-deformed and deformed specimens were not significantly different Therefore, deformation of single use files may not affect the efficiency of the files. However, clinically, a rotary file will be replaced immediately after deformation is observed to prevent a broken instrument and root canal obstruction.

There are potential problems when a rotary file breaks during retreatment. Broken rotary files impede root canal filling removal and compromise intraradicular infection removal. Moreover, broken instrument retrieval can result in increased chair-time, cost, and tooth structure loss that may reduce the fracture resistance of the root. The specimens with broken rotary files were excluded from the analysis because including the root canal filling volume and retreatment time of these specimens in the analysis may cause overestimation of both data and result in underestimating the efficacy and efficiency of the rotary files.

Achieving high efficacy in retreatment in the apical-third of the root canal is vital to achieve a satisfactory root canal retreatment outcome. Intraradicular infection in the apical area can lead to periapical inflammation. This is because the apical foramen is the main communication pathway between the root canal system and the periapical tissue. Although the ratio of the apical-third to the whole canal volume in this study was only 11%, dentists should choose NiTi rotary files that can efficiently retreat the apical-third area.

The remaining root canal filling material can impede intra-dentinal tubule disinfection because disinfection using chemo-mechanical instrumentation and medication is not possible in the area that is covered by the remaining root canal filling. The remaining intraradicular infection beneath the root canal filling can cause periapical inflammation through apical leakage [35]. However, entombment of the bacteria in the root canal by appropriate root canal obturation at the working length can prevent periapical inflammation from intraradicular infection [36].

The results of the present study suggest that X2 with continuous rotation and RB files can be used with high efficacy and efficiency as a single file in curved root canal retreatment. Considering patient comfort and operator fatigue, an efficient retreatment technique should be used. To achieve the maximum efficacy and efficiency in curved root canal retreatment, single use of a rotary file in only one molar is recommended. Multiple use of a rotary file can reduce the cyclic fatigue resistance of the rotary file [37, 38].

The strength of the study is that the specimens were prepared by a single operator using the same technique and the curvature and pre-retreatment filling volume in each group was similar. The Post-Micro-CT analysis was done by another operator who knew only the specimen number, but not the NiTi rotary file group to which it belonged. Moreover, the aim of the current study was to compare the efficacy and efficiency of different NiTi rotary instruments using different motions in retreating curved root canals. Thus, using one file per root canal can indicate the true efficacy and efficiency of the NiTi rotary file. The results from the ProTaper NEXT group using continuous rotation and adaptive motion suggest that using continuous rotation is more efficacious and efficient than adaptive motion in curved root canal retreatment when using the same rotary file system. Single file retreatment using X2 with continuous rotation or RB files in a small curved root canal can be done with high efficacy, efficiency, and cost-effectiveness.

The limitation of the current study was that the specimens used were only the mesial canals of mandibular molars. The cleanliness of the retreatment procedure in the present study may be due to the approximate sizes of the NiTi rotary files and root canals. Therefore, the results of this study can only be generalized to retreating teeth with small curved root canals, such as the mesial root of the mandibular molars and the buccal roots of the maxillary molars [21, 22, 39]. However, X2 with continuous rotation and RB files can be used for penetrating to the working length of a curved canal in the original canal path without complications. Therefore, X2 with continuous rotation and RB files can be used for creating the path in large or oval root canals up to the working length, and then a bigger rotary file or sequent rotary files should be used for complete root canal filling removal. Another limitation of the study was that the operator could not be blinded to the retreatment procedures due to the different appearances of the rotary files. Our results demonstrated that the NiTi rotary files used in small curved root canals were at risk of breakage.

A prospective research investigation could explore the efficacy of a retreatment procedure involving hand files with solvent to create a glide path, followed by NiTi rotary, to reduce the risk of rotary file breakage when negotiating gutta percha filling in small curved root canals.

## Conclusion

Based on our results, X2 with continuous rotation and RB files can be used with high efficacy in curved root canal retreatment. Furthermore, continuous rotation has been found to be more efficacious and efficient than adaptive motion when using the same NiTi rotary file. Single file retreatment can be performed in small canals with high efficacy and in a cost-effective manner, with less time consumed.

## Data Availability

The data that support the findings of this study are available from the corresponding author upon reasonable request.

## List of abbreviations

µCT: Micro-Computed Tomography
NS-ReTx: Non-surgical root canal retreatment
NiTi: Nickel-titanium
NaOCl: Sodium hypochlorite
EDTA: Ethylenediamine tetraacetic acid
DOM: Dental operating microscope
RB: Reciproc® *Blue* system
VR: VDW.ROTATE retreatment system
PTNc: ProTaper NEXT X2 continuous rotation
PTNa: ProTaper NEXT X2 adaptive motion
ICC: Intraclass correlation coefficient
MD: Mean difference
CI: Confidence interval
